# Improvement of Fibromyalgia by Immunoglobulin/Histamine Complex Therapy for Chronic Urticaria and Allergic Rhinitis: A Case Report

**DOI:** 10.1002/ccr3.73248

**Published:** 2026-08-09

**Authors:** Hyuk Soon Kim, Yunyoung Nam, Jeong Eun Sohn, Geunwoong Noh

**Affiliations:** ^1^ Department of Biomedical Sciences, College of Natural Science and Department of Health Sciences The Graduate School of Dong‐A University Busan Republic of Korea; ^2^ Department of Anesthesiology Cheju Halla General Hospital Jeju‐si Jeju Republic of Korea; ^3^ Department of Allergy and Clinical Immunology Cheju Halla General Hospital Jeju‐si Jeju Republic of Korea

**Keywords:** chronic urticaria, fibromyalgia, histamine, Histobulin, immunoglobulin/histamine complex

## Abstract

Fibromyalgia (FM) is a chronic pain syndrome characterized by widespread musculoskeletal pain, often accompanied by fatigue, sleep disturbance, and cognitive symptoms. Although several treatment options are available, no curative therapy has been established. Histobulin is a histamine‐fixed immunoglobulin preparation developed to regulate serum histamine levels through histaminopexy. It has been reported to have therapeutic effects in allergic rhinitis, bronchial asthma, chronic urticaria, and atopic dermatitis. We report a patient with FM and concomitant chronic urticaria and allergic rhinitis who experienced improvement in FM after Histobulin therapy. A 39‐year‐old Korean woman presented with a 6‐year history of chronic urticaria, allergic rhinitis with rhinorrhea, and widespread musculoskeletal pain. She had previously been diagnosed with FM and had received oral treatment for pain for 13 months. At the first visit, her widespread pain index (WPI) was 9 and her symptom severity scale (SSS) score was 12. Histobulin therapy was initiated primarily for chronic urticaria and allergic rhinitis. During treatment, she reported reductions in widespread pain, finger stiffness, and insomnia, as well as improved daily functioning and reduced use of rescue analgesics. After six injections, the patient reported marked reductions in FM symptoms and in symptoms of urticaria and rhinitis. No recurrence was reported during the subsequent 3‐week follow‐up period. FM symptoms improved after Histobulin therapy initiated for chronic urticaria and allergic rhinitis. This observation suggests that modulation of histamine‐related or immune pathways may be associated with clinical improvement. However, causality cannot be established from a single case report, and further controlled studies are required.

## Key Clinical Message

Fibromyalgia was improved after immunoglobulin/histamine complex therapy administered for chronic urticaria and allergic rhinitis. This temporal association is hypothesis‐generating and warrants evaluation in controlled studies; causality cannot be inferred from a single case.

**Abbreviations**
Df: *Dermatophagoides farinae*
Dp: *Dermatophagoides pteronyssinus*
FM: fibromyalgia
IHC: immunoglobulin/histamine complex

## 1 Introduction

Fibromyalgia (FM) is a chronic pain syndrome characterized by widespread musculoskeletal pain and commonly accompanied by fatigue, sleep disturbance, cognitive symptoms, anxiety, and depressive symptoms [1, 2, 3, 4, 5, 6]. The etiology of FM remains incompletely understood. Prevalence estimates vary across populations, but FM is more common in women than in men [3, 4]. Although multiple pharmacological and nonpharmacological treatments are available, no curative therapy has been established [2]. Complementary approaches, including acupuncture, are also used for symptom management [7].

Histobulin (Green Cross PD, Korea) is a histamine‐fixed immunoglobulin preparation comprising 0.15 μg of histamine dihydrochloride and 12 mg of IgG [8]. It was developed to regulate serum histamine levels via histaminopexy [9]. Histobulin has been reported to induce remission in chronic spontaneous urticaria [10]. We report a patient with FM and concomitant chronic urticaria and allergic rhinitis who experienced improvement in FM symptoms following immunoglobulin/histamine complex therapy.

## 2 Case History and Examination

A 39‐year‐old Korean woman presented to the Department of Allergy and Clinical Immunology at Cheju Halla General Hospital with a 6‐year history of chronic urticaria and rhinorrhea. She also reported chronic widespread pain involving multiple body regions. Her medical history included an anaphylactic reaction to contrast media 9 years earlier and urticaria following diazepam administration 4 years earlier. She had a history of smoking and heavy alcohol consumption.

She had previously been diagnosed with FM and had received celecoxib and eperisone hydrochloride for pain management for 13 months. She also had a history of depression treated with amitriptyline. Her older sister had ankylosing spondylitis.

She reported long‐standing fatigue, prolonged sleep duration, arthralgia, back pain, and myalgia. Her symptoms had persisted for 6 years, and pain was present in at least four of five body regions. Her WPI and SSS scores were 9 and 12, respectively; therefore, she met the 2016 revised ACR diagnostic criteria for FM [11].

The evaluation of chronic urticaria and allergic rhinitis included a complete blood count with differential, measurements of serum eosinophil cationic protein and total IgE, allergen‐specific IgE testing using the multiple allergosorbent test (MAST), and skin prick testing (Table 1A–C). Serum immunoglobulin concentrations and IgG subclasses were also assessed (Table 1A). Because of the chronic widespread pain and joint symptoms, rheumatologic and radiologic evaluations were performed (Table 1D).

**TABLE 1:** Baseline laboratory, allergen sensitization, and radiologic findings. (A) Laboratory findings. (B) Allergen‐specific IgE levels measured using the multiple allergosorbent test (MAST; Green Cross PD, Korea). (C) Skin prick test results. (D) Radiologic findings. For MAST, values < 0.35 IU/mL were considered negative.

(A) Laboratory findings			
Classification	Test items	Results (grade)	Normal range (unit)
Allergy	Blood eosinophil fraction	0.6	0–5 (%)
	Blood basophil fraction	0.3	0.0–1 (%)
	Serum eosinophil cationic protein	10	0–24 (ng/mL)
	Serum total IgE	11	350 > = (IU/mL)
Immunology	Serum IgA	59	70–400 (mg/dL)
	Serum IgG	905	700–1600 (mg/dL)
	Serum IgG1	452.5	382.4–928.6 (mg/L)
	Serum IgG2	324	241.8–700.3 (mg/L)
	Serum IgG3	41.6	21.8–176.1 (mg/L)
	Serum IgG4	14.4	3.9–86.4 (mg/L)
	Serum IgM	102	40–230 (mg/dL)
	Serum IgD	0.68	0.77–13.2 (mg/dL)
	C3	101	90–180 (mg/dL)
	C4	26	10–40 (mg/dL)
	HBs Antigen	Negative	
	HBs Antibody	Positive	
Rheumatology	Anti nuclear Ab (ANA)	1:40>	1:40>
	Anti‐cyclic citrullinated peptide (CCP) Ab	8>	8 > (U/mL)
	Rheumatoid factor (RF)	8.7	0.0–20.0 (IU/mL)
	C‐reactive protein (CRP)	0.04	0.00–0.30 (mg/dL)
	HLA B27 typing	Negative	
	Anti‐SSa/Ro antibody	Negative	
	Anti‐SSb/La antibody	Negative	
Endocrinology	FSH	7.59 WNL	(mIU/mL)
	LH	3.69 WNL	(mIU/mL)
	Testosterone	0.1 > Low	
	E2	59.3 WNL	pg/mL
	Prolactin	7.28 WNL	Ng/mL
	TSH	1.670	0.279–4.200 (mIU/L)
	T3	1.14	0.80–2.00 (ng/mL)
	Free T4	1.030	0.930–1.700 (ng/dL)
	Cortisol	2.70	(ug/dL)
	HGH	0.58	0.10–8.00 (ng/mL)
	ACTH	5.24	7.20–63.30 (pg/mL)
	IGF‐1	184 WNL	(ng/mL)
Psychologic tests	Beck depression inventory (BDI)‐2	47 (Severe)	Normal ≤ 13, mild 14–19, moderate 20–28, and severe 29–63
	Beck Hopelessness Scores	15 (Severe)	Normal < 4, mild 4–8, moderate 9–14, and severe 15–20
	State–Trait Anxiety Inventory (STAI) State	70 (Severe)	Normal ≤ 51, mild 52–56, moderate 57–61, and severe 62–80
	State–Trait Anxiety Inventory (STAI) Trait	76 (Severe)	Normal ≤ 53, mild 54–58, moderate 59–63, and severe 64–80

(B) MAST (IU/mL)			
MAST allergens	Result	Allergens	Result
*Alternaria alternata*	0.35>	Reed	0.35>
*Aspergillus fumigatus*	0.35>	Japanese hop	0.35>
*Penicillium notatum*	0.35>	Acacia	0.35>
*Cladosporium herbarum*	0.35>	Pine	0.35>
Cockroach	0.35>	Poplar mix	0.35>
House dust mites	0.35>	Sycamore	0.35>
*Dermatophagoides pteronyssinus*	0.35>	Ash mix	0.35>
*Dermatophagoides farinae*	0.35>	Oxeye daisy	0.35>
Dog	0.35>	Dandelion	0.35>
Cat	0.35>	Russian thistle	0.35>
Birch‐Alder mix	0.35>	Goldenrod	0.35>
Mugwort	0.35>	Pigweed	0.35>
Short Ragweed	0.35>	Crab	0.35>
Sallow willow	0.35>	Shrimp	0.35>
Orchard grass	0.35>	Mackerel	0.35>
Bermuda grass	0.35>	Soybean	0.35>
Timothy grass	0.35>	Hazelnut	0.35>
Sweet vernal grass	0.35>	Peach	0.35>
Rye pollen	0.35>	Milk	0.35>
White oak	0.35>	Egg white	0.35>
Japanese cedar	0.35>		

(C) Skin prick test (grade)			
Allergens	Result	Allergens	Result
Alternaria alternata	—	Beef	—
*Aspergillus fumigatus*	—	Pork	—
*Aspergillus niger*	—	Cod	—
*Candida albicans*	—	Oyster	—
Cladosporium	—	Salmon	—
*Penicillium chrysogenum*	—	Prawn	—
German cockroach	—	Mackerel	—
*Dermatophagoides pteronyssinus*	—	Tuna	—
*Dermatophagoides farinae*	—	Almond	—
Dog	—	Peanut	—
Cat	—	Bean	—
Gray alder. Silver birch mix	—	Carrot	—
Grass mix	—	Cabbage	—
Mugwort	—	Walnut	—
Short Ragweed	—	Maize	—
Black willow pollen	—	Peach	—
Orchard	—	Tomato	—
Bermuda grass	—	Black pepper	—
Timothy	—	Spinach	—
English plantain	—	Wheat	—
English Rye grass	—	Rabbit	—
Holm oak	—	Kapok	—
Japanese cedar	—	Hop	—
Latex	—	*F acacia*	—
Milk	—	Pine	—
Egg	—	Poplar	—
Chicken	—		

(D) Radiologic findings		
Radiologic study	Test items	Interpretation
Simple X‐ray	Hand AP both	Diffuse OA in the DIP Joints
	Hand oblique both	No remarkable body abnormalities
	S‐I joint both	Unremarkable
	L‐spine	Back sprain
Whole body bone scan	Posterior view	Increased uptake in sacrum

*Note:* Skin prick test responses were graded as follows: 0, no reaction; 1+, a wheal larger than the negative control but less than half the size of the histamine control wheal; 2+, a wheal at least half the size of the histamine control wheal; 3+, a wheal equal to or larger than the histamine control wheal; and 4+, a wheal at least twice the size of the histamine control wheal.

Most allergy‐related laboratory measures, including the eosinophil fraction, serum eosinophil cationic protein, and total IgE, were within their respective reference ranges. No allergen sensitization was detected by MAST or skin prick testing (Table 1B,C). Immunologic testing revealed low IgA and low IgD levels. Rheumatoid factor was within the reference range, and the anti‐CCP antibody test was negative. The available clinical, laboratory, and radiologic findings did not support a diagnosis of rheumatoid arthritis or another systemic autoimmune disease.

The radiologic evaluation did not reveal findings diagnostic of inflammatory arthritis. Diffuse osteoarthritic changes in the distal interphalangeal joints and increased radiotracer uptake in the sacral region on whole‐body bone scintigraphy were noted (Table 1D).

A clinical psychologist assessed sleep disturbance and psychological symptoms using the Beck Depression Inventory‐II (BDI‐II), Beck Hopelessness Scale (BHS), and State–Trait Anxiety Inventory (STAI) [12]. The BDI‐II, BHS, STAI‐State, and STAI‐Trait scores were 47, 15, 70, and 76, respectively, all within the severe range.

The severity of chronic spontaneous urticaria was assessed using the Urticaria Severity Score developed by Jariwala et al., yielding a total score of 93 [13]. Pain intensity was evaluated using the numeric rating scale (NRS) [14, 15].

## 3 Conclusions and Results

**FIGURE 1:**
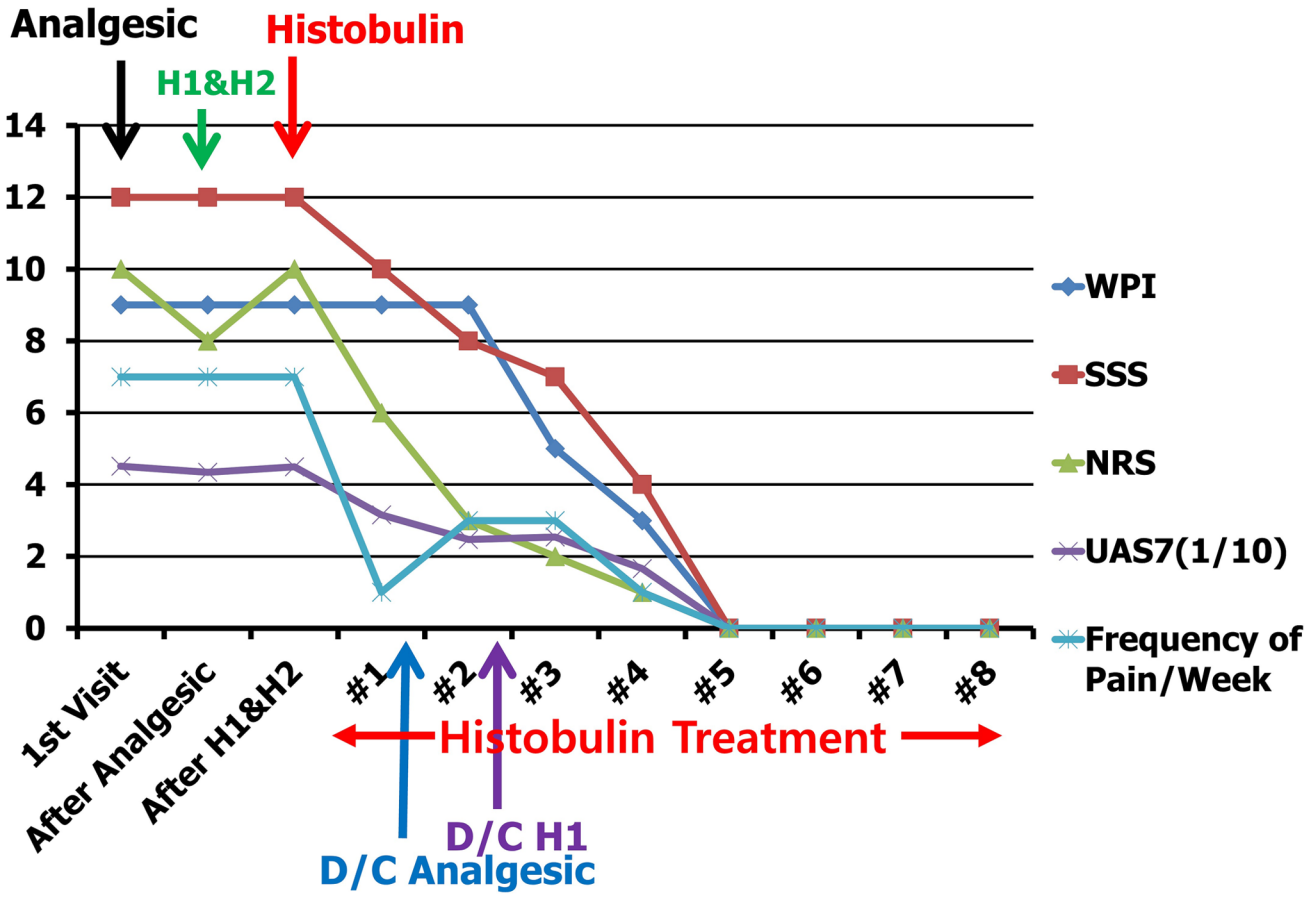
Longitudinal course of fibromyalgia symptoms and allergic manifestations during treatment. Clinical measures included the WPI, SSS, NRS, UAS7, weekly pain frequency, and rescue analgesic use. At the first visit, Synerjet was prescribed as rescue analgesia, but pain recurred daily. During the second week, levocetirizine and cimetidine improved rhinitis and urticaria, whereas widespread pain persisted. Histobulin therapy for chronic urticaria and allergic rhinitis was initiated in the third week; the red arrow indicates the first injection. During subsequent treatment, the patient reported reductions in widespread pain, finger stiffness, insomnia, and rescue analgesic use. No recurrence of chronic urticaria, allergic rhinitis, or FM symptoms was reported during the 3‐week follow‐up period. UAS7 values were divided by 10 for graphical scaling. D/C, discontinued; FM, fibromyalgia; H1, histamine H1‐receptor antagonist; H2, histamine H2‐receptor antagonist; NRS, numeric rating scale; SSS, symptom severity scale score; UAS7, 7‐day urticaria activity score; WPI, widespread pain index.

At the first visit, half a tablet of Synerjet (acetaminophen 162.5 mg/tramadol hydrochloride 18.75 mg; Samjin Pharmaceutical Co. Ltd., Korea) was prescribed as rescue analgesia. Although the medication provided temporary pain relief (Figure 1), pain recurred daily. During the second week, Synerjet was discontinued, and levocetirizine and cimetidine were initiated. Rhinitis and urticaria improved, whereas widespread pain persisted.

During the third week, immunoglobulin/histamine complex (IHC) therapy with Histobulin was initiated (Histobulin #1) for chronic spontaneous urticaria and allergic rhinitis. Each 2‐mL ampule of Histobulin contained 12 mg of human immunoglobulin and 0.15 μg of histamine dihydrochloride (Green Cross PD, Korea). One ampule was administered subcutaneously in the deltoid region once weekly. The patient was instructed to use Synerjet only for severe pain.

After the first Histobulin injection, the patient reported reductions in widespread pain and finger stiffness and did not require rescue analgesia with Synerjet. Over the subsequent weeks, she reported gradual reductions in finger pain, ankle and knee heaviness, back pain, insomnia, and facial eruptions. A transient mild worsening of finger stiffness and pain was reported after the third injection. After the sixth injection, the patient reported marked reductions in FM symptoms and allergic manifestations. No recurrence of urticaria or rhinitis symptoms or of FM symptoms was reported during the subsequent 3‐week follow‐up period. Longitudinal changes in WPI, SSS, NRS, urticaria activity, weekly pain frequency, and rescue‐medication use are summarized in Figure 1.

The improvement in FM occurred in temporal association with IHC therapy administered for chronic urticaria and allergic rhinitis. However, this single‐case observation in a patient with concomitant allergic conditions and immunologic abnormalities does not establish therapeutic efficacy or causality.

## 4 Discussion

In this case, the patient reported improvement in fibromyalgia pain and associated symptoms after IHC therapy with Histobulin, which had been initiated primarily for chronic urticaria and allergic rhinitis. The temporal association between IHC therapy and symptom improvement raises the possibility that histamine‐related or immune mechanisms may have contributed to the observed clinical course. However, this single‐case observation cannot establish causality.

Current management of FM includes both pharmacological and nonpharmacological approaches, yet many patients continue to experience persistent symptoms despite treatment [14]. Accordingly, carefully interpreted clinical observations may help generate hypotheses regarding additional mechanisms or therapeutic strategies.

Previous reports have described clinical improvement following IHC therapy in several inflammatory or allergic conditions [16, 17, 18]. Although these observations do not establish efficacy across conditions or a general analgesic effect, they provide a rationale for investigating whether histamine‐related or immune mechanisms may contribute to pain in selected clinical contexts.

Several studies have suggested possible links among allergic mechanisms, mast cells, histamine signaling, and chronic pain conditions, including FM [19, 20, 21]. Histamine‐related dietary responses and mast‐cell abnormalities have also been reported in patients with FM. However, these findings remain preliminary and do not establish histamine signaling as a primary driver of FM. Mast‐cell‐mediated neuroimmune signaling has likewise been proposed as a contributor to pain in FM, although this hypothesis remains unproven [22].

Histamine receptors, particularly H3 and H4 receptors, have been implicated in the modulation of neuropathic pain [23], although the relevance of these findings to FM remains uncertain. Histaminopexy has been proposed as a mechanism through which histamine‐fixed immunoglobulin preparations may exert anti‐allergic effects [24]. Modulation of histamine‐related pathways may therefore represent one possible explanation for the clinical course observed in this patient; however, this interpretation remains speculative.

Inflammatory and immune mechanisms have also been proposed to contribute to FM pathophysiology in some patients, although altered central pain processing remains a well‐established feature of the disorder [22, 25, 26]. Reports of altered cytokine and chemokine profiles further suggest that immune‐related pathways may contribute to symptom expression in certain patient subgroups [27, 28].

In addition to histaminopexy, anti‐inflammatory effects have been proposed as a potential mechanism of IHC therapy [18, 29]. If such effects occurred in this patient, they may have contributed to the observed symptom improvement. However, this possibility cannot be confirmed in the absence of serial inflammatory biomarkers or dechallenge–rechallenge evidence.

Autoimmune features have been described in subsets of patients with FM, particularly in those with comorbid rheumatologic disease [30, 31]. In the present case, however, rheumatoid factor was within the reference range, the anti‐CCP antibody test was negative, and the available clinical and radiologic findings did not support a diagnosis of rheumatoid arthritis or another systemic autoimmune disease. Low IgA and IgD levels were noted, but their clinical relevance remains uncertain. Moreover, the dose and formulation of Histobulin differ from those of conventional intravenous immunoglobulin therapy [32], precluding conclusions regarding an immunoglobulin‐mediated mechanism.

Because pain is subjective and FM symptoms may fluctuate over time, standardized longitudinal measures are important when interpreting clinical changes. In this case, the available measures included the WPI, SSS, NRS, pain frequency, urticaria activity, and rescue analgesic use, as summarized in Figure 1. The reduction in rescue analgesic use provides additional information regarding the clinical course but should be interpreted cautiously because the assessment was uncontrolled and involved a single patient.

Several limitations should be considered. This was an uncontrolled single‐case observation with a short follow‐up period. The fluctuating nature of FM symptoms, placebo effects, regression to the mean, spontaneous improvement, and the potential influence of concurrent or recent treatments cannot be excluded. In addition, not all clinical outcomes were assessed at every time point, and no serial mechanistic biomarkers were available. Therefore, the observed improvement should be regarded as hypothesis‐generating rather than as evidence of therapeutic efficacy.

This case suggests that documenting allergic conditions and immunologic abnormalities may be informative when evaluating patients with FM symptoms. Controlled studies with standardized outcome measures and longer follow‐up periods are needed to determine whether the observed temporal association is reproducible and clinically meaningful.

## Author Contributions

**Hyuk Soon Kim:** conceptualization. **Yunyoung Nam:** conceptualization. **Jeong Eun Sohn:** conceptualization. **Geunwoong Noh:** writing – original draft.

## Funding

The authors have nothing to report.

## Ethics Statement

This case report was approved by the Institutional Review Board of Cheju Halla General Hospital (IRB No. 2020‐M07‐01).

## Consent

Written informed consent was obtained from the patient for publication of this case report and any accompanying images. The written consent is available for review by the editor upon request.

## Conflicts of Interest

The authors declare no conflicts of interest.

## Data Availability

The authors have nothing to report.
